# A comparison of the effect of Swedish massage with and without chamomile oil on labor outcomes and maternal satisfaction of the childbirth process: a randomized controlled trial

**DOI:** 10.1186/s40001-022-00901-x

**Published:** 2022-11-25

**Authors:** Fatemeh Eskandari, Parvaneh Mousavi, Mahboubeh Valiani, Saeed Ghanbari, Mina Iravani

**Affiliations:** 1 M.Sc. student in midwifery, Department of Midwifery, School of Nursing and Midwifery, Reproductive Health Promotion Research Center, Ahvaz Jundishapur University of Medical Sciences, Ahvaz, Iran; 2 Department of Midwifery and Reproductive Health, School of Nursing and Midwifery, Reproductive Health Promotion Research Center, Ahvaz Jundishapur University of Medical Sciences, Ahvaz, Iran; 3 Department of Midwifery and Reproductive Health, School of Nursing and Midwifery, Reproductive Sciences and Sexual Health Research Center, Isfahan University of Medical Sciences, Isfahan, Iran; 4 Department of Bioststistics and Epidemiology, School of Public Health, Ahvaz Jundishapur University of Medical Sciences, Ahvaz, Iran; 5 Department of Midwifery and Reproductive Health, School of Nursing and Midwifery, Reproductive Health Promotion Research Center, Ahvaz Jundishapur University of Medical Sciences, Ahvaz, Iran

**Keywords:** Swedish massage, Chamomile oil, Delivery outcomes, Labor

## Abstract

**Purpose of study:**

Massage is widely used as a traditional technique during labor and delivery. The aim of this study was to evaluate the effect of Swedish massage with and without chamomile oil on delivery outcomes.

**Methods:**

The present study was a randomized clinical trial on 154 primiparous pregnant women who were selected by random sampling method and divided into 3 groups: massage with chamomile oil (*n* = 53), massage without chamomile oil (*n* = 51), and control group (*n* = 50). Data collection tools included demographic and delivery information questionnaire, Visual Analogue Scale (VAS), maternal satisfaction with delivery scale, and Partograph form. In the two intervention groups, Swedish massage techniques (i.e., Effleurage, Petrissage, Vibration, and Superficial stroke) were performed with and without the use of chamomile oil since the active phase of labor (5 cm dilatation), while the control group received only routine labor care during labor.

**Results:**

Swedish massage with chamomile oil significantly reduced the labor pain score, reduced the length of active phase and the second stage of labor, and increased the score of maternal satisfaction with the labor process (*P* < 0.001). In addition, there was a significant relationship between the type of study groups and the type of delivery (*P* < 0.043).

**Conclusion:**

The results of the study showed that using Swedish massage techniques with or without chamomile oil can improve labor outcomes. Swedish massage using chamomile oil was associated with better results compared to the same massage without using chamomile oil.

*Clinical trial code* IRCT20200513047430N1.

## Introduction

The mechanism and physiology of delivery entail a natural, spontaneous and non-interventional process that has been performed physiologically for many years [1]. As a critical and important experience in the life of every woman, delivery has several physical, emotional, social, physiological, cultural and psychological dimensions [2]. In most cases, the best mode of delivery for the mother is spontaneous vaginal delivery, which involves a physiological and natural process offering the mother and baby with many benefits [3].

Duration of labor is one of the factors affecting pregnancy outcomes as well as maternal and neonatal complications. Prolonged delivery poses the risk of fetal or neonatal death, suffocation, infection, and neurological and physical damage to the neonate. In addition, the mother is susceptible to postpartum hemorrhage and infection as well as emotional problems due to anxiety, insomnia, and fatigue [4].

In recent years, the demand for cesarean section by pregnant women has been on the rise, which not only leads to increased complications of childbirth, but also imposes additional financial burdens on families, government and insurance organizations. By educating pregnant women, they can be involved in decision making to manage their pregnancies [1].

However, due to the painful nature of the delivery process, humans have long sought to find a way to reduce pain as one of the consequences of childbirth. For example, 59% of the causes of cesarean section are due to fear of normal labor pain, which indicates that fear of normal labor pain can lead to an increase in cesarean section rates in pregnant women [5].

Pain is the most common and unavoidable component of natural childbirth [6]. Labor pain is one of the most severe pains one can experience [7]. The combination of severe pain, anxiety, and stress during labor and delivery leads to increased secretion of catecholamines and decreased uterine activity, and thus prolonged labor [8]. The duration of labor pains and the resulting anxiety also interfere with the normal function of the respiratory system, the endocrine system, and blood circulation, which ultimately increases the risk of difficult labor and failure in normal progression of labor. In addition, it increases the rate of intervention, instrumental delivery, and birth of babies with low Apgar scores. Prolonged delivery also increases the risk of infection, uterine atony, postpartum hemorrhage, excessive fatigue, psychological trauma, and anxiety in the pregnant mother [9].

Research shows that about 60% of primiparous women and 40% of multiparous women suffer from severe and unbearable pain during childbirth [10]. One of the important factors to increase maternal satisfaction with childbirth is the use of methods to reduce labor pain [11]. These methods are divided into two general categories: non-pharmacological (psychotherapy, hypnosis, massage, acupuncture, acupressure, aromatherapy, music, etc.) and pharmacological (systemic drugs, inhaled analgesics, and general or local anesthesia) [12–14].

Massage is defined as a systematic form of stroking the soft tissues of the body with hands for therapeutic purposes such as relieving pain and increasing comfort and well-being of patients [15]. Massage is used as an old technique in childbirth, and by reducing the secretion of adrenaline and noradrenaline hormones and increasing endorphins and oxytocin secretion, it reduces labor pain and thus shortens the duration of labor by increasing uterine contractions [16]. Swedish massage is a set of simple massage therapy techniques designed to put pressure on muscles and bones, and as vivo massage, it is designed to return blood to the heart and is a standard treatment [17].

The use of aromatic vegetable oils has been common in Egypt and India for thousands of years and is still recommended today by the US National Council of State Boards of Nursing (NCSBN) [18]. In addition, aromatic oils are nowadays being used for painless natural childbirth and cesarean delivery [19]. During childbirth, oil massage promotes various stages of labor, and one of the purposes of using oil massage is to reduce anxiety and fear and help the mother get relaxed [20]. Iranian traditional medicine recommends massaging the woman's body especially the middle part of the body (back and abdomen) with oil in order to facilitate childbirth and help women to adapt to labor pains [21].

One of the vegetable oils that can be used for this purpose is chamomile flower oil. This plant contains 120 types of chemical compounds, the main components of which include alpha bisabolol, bisabolol oxide, spiruthers, camazoline, flavonoids, azoline, and coumarin. Chamomile is used in the treatment of skin diseases such as psoriasis, eczema, and acne. It is also used to ease fever and treat bronchitis, coughs, and colds. In addition, chamomile has been shown to have analgesic, antispasmodic, anti-inflammatory, and antimicrobial effects [22]. German chamomile essential oil has strong anti-inflammatory and analgesic effects due to its camazoline content. Rafiei et al. examined the effect of massage with a combination of lavender and chamomile aromatic oils on the severity of underlying pain in patients with burns. Their results showed that massage with aromatic chamomile oil is effective in reducing pain in these patients due to its analgesic properties [23].

Given the important role of complementary medicine and non-pharmacological methods of reducing pain in the delivery process [24] and the scant research on the effect of performing Swedish massage techniques using chamomile oil during labor on the consequences of childbirth, the present study aimed to determine the effect of Swedish massage with (out) chamomile oil on the consequences of childbirth and the degree of satisfaction of primiparous women with the labor process.

## Objectives

The aim of this study was to investigate the effect of Swedish massage with chamomile oil on delivery outcomes and maternal satisfaction during labor in primiparous women.

### Method

The present study was a controlled randomized clinical trial conducted from 2020–09-10 to 2021–04-19 in 22 Bahman Hospital of Masjed Soleiman, Khuzestan Province, southwest of Iran.

Inclusion criteria were: primiparous women aged between 18 and 35 years, giving consent to participate in the study, having normal body mass index, singleton pregnancy, being at gestational age between 37 and 41 weeks, having reactive nonstress test (NST), uncomplicated pregnancy, 5 cm cervical dilatation, and experiencing 3 contractions per 10 min. Exclusion criteria were: mental health problems, chronic diseases (heart, kidney, respiratory, diabetes and hypertension), skin diseases (allergies, eczema and inflammation), abnormal fetal heart rate pattern, placental abruption, umbilical cord prolapse, prenatal consumption of drugs and analgesics, and allergies to vegetable oils.

Based on a study by Gallo et al. [25], assuming a change in the mean ± standard deviation score of cervical dilatation from 5.7 ± 1.5 to 6.8 ± 1.6 after of massage therapy in the control and intervention groups, respectively, with a *ɑ* = 0.05 and a statistical power of 90%, Effect size of 0.70, the sample size was calculated to be 43 women for each group using G-power software. Given the possible 20% attrition rate, 53 women were allocated to each of intervention and control groups.

All participants completed and signed written informed consent form before commencement of the study. The women were selected according to the inclusion and exclusion criteria and were randomly divided into one of the three groups using numbered cards, namely the Swedish massage group with chamomile oil, the Swedish massage group without chamomile oil, and the control group. Then 159 cards were prepared according to the sample size and the letters A, B or C were written on each card. The cards were placed in a box.

Any of the participants randomly selected one card, and the chosen cards were removed from a deck of cards. Pregnant mothers who chose A cards entered the Swedish massage group using chamomile oil (intervention group 1), those choosing B cards entered the Swedish massage group without using chamomile oil (intervention group 2), and participants choosing C cards received only the routine care (control group).

Thus, none of the mothers and researcher knew the type of intervention prior to the commencement of the intervention. Due to the nature and type of intervention, there was no possibility of blinding the pregnant mothers or the researchers. In this study, the intervention was performed by the researcher, but in order to make bias less likely, the person evaluating the results was not a member of the research team.

At the outset of the study, the initial participants were briefed on the objectives of the research and the way the intervention was going to be carried out. In order to observe ethical considerations, women who were willing to participate were asked to sign a written informed consent. Also, the participants were assured that their information would remain completely confidential and that they could withdraw from the study any time they desired.

Pregnant mothers with 5 cm cervical dilatation were included in the study. Massage techniques were started from the active phase of labor (5 cm dilatation). The expectant mother was placed in a sitting or half-sitting position on a chair or leaning on a delivery ball, and the masseuse stood in front of the mother massaging her arms and legs. To massage her back, the mother sat on a chair with her face facing the backrest of the chair, or she would kneel on the delivery ball. In this case, the masseuse stood behind the mother and performed the massage.

To start from a cervical dilation of 5 cm to 8 cm, a superficial stroke of the back was used for the shoulders, arms and legs, plus a spinal Effleurage, each lasting for 10 min. As soon as 8 cm dilatation started, Petrissage or strong stroke massage was applied to the lumbar and dorsal areas (the B-shaped and heart-shaped areas), Petrissage of the shoulders and both sides of the spine was applied (in a walking and spiral form), along with vibration and sacrum compressions, each lasting for 10 min, and this was done until 10 cm dilation and complete opening of the cervix. In each of these two stages (i.e., dilatation of 5 cm to 8 cm and dilation of 8 cm to 10 cm) at least 3 massage rounds (6 times in total) were performed once every 20 min, each lasting for 10 min. The interval between the massage rounds was proportional to the mother's progress. After complete dilatation of the cervix, due to the mother's being in the lithotomy position and lack of access to the sacrum, it was not possible to perform the massage. In each stage, according to the severity of pain and emotional needs of the mother, the type of massage was selected. That is, in dilatation of 8 to 10 cm, because the pain was more severe, the massage was performed with more focus on the lower back and the sacrum to reduce the severity of the mother's pain in a more favorable way.

The five Swedish massage techniques including Effleurage (superficial and long strokes) in the head, arms, legs and spine, Petrissage (strong stroke of the thoracic and lumbar regions and shoulders), vibration (rapid muscle vibration) and compression (compression massage) were done on the sacrum.

In group A, Swedish massage was performed using odorless chamomile massage oil prepared by Ganjineh Osareh Tabiat (Noshad) Pharmaceutical Company, Tehran, Iran. Before the intervention, to check the women's insensitivity to the oil used, a sensitivity test was performed by rubbing some oil on the areas of the body with healthy skin. A stopwatch was used to measure the duration of the massage. After washing her hands, the masseuse poured five cc of chamomile oil into her palms, and by rubbing them on each other, the oil was warmed up. Upon the start of the active phase of labor (5 cm dilatation), the massage was started. In case it was necessary to use oxytocin during labor, the amount of oxytocin intake was calculated and recorded in milliunits. The mother was asked to close her eyes during the massage and to focus on the massage. The room where the massage was performed was adequately lit and properly ventilated to create a private and cozy environment for the participants in the best possible way.

In all three groups (Group A: Swedish massage using chamomile oil, Group B: Swedish massage without chamomile oil, and Group C: control group) pain intensity assessment was done in 4 stages (5 cm, 8 cm, 10 cm dilations, and immediately after delivery) by an assessor in the absence of the researchers. Maternal anxiety and satisfaction with the delivery process were measured using a questionnaire and self-report method in 5 cm dilatation (before intervention) and immediately after delivery. Also, the progress of delivery, length of the active phase and the second stage of labor, the amount of injectable oxytocin (in terms of milliunits) and the type of labor were recorded by the researcher in the partograph form and the delivery information checklist.

The data collection tools in this research included: a demographic questionnaire, delivery information checklist, Visual Analogue Scale, delivery satisfaction questionnaire, and partograph form.

The Visual Analogue Scale (VAS), which scores pain intensity from 0 to 10, was used to assess pain intensity. This scale has been used in various studies and its reliability and validity have been confirmed [26].

To measure maternal satisfaction, the delivery process satisfaction questionnaire developed in 2017 by Mahmoudi et al. at Isfahan University of Medical Sciences was used. The validity and reliability of this questionnaire had already been determined. A score of 65 and above indicated high satisfaction, a score of 45–64 indicated moderate satisfaction, and a score of 44 and below indicated low satisfaction [27].

In this study, SPSS version 20 was used to analyze the data using descriptive statistics (mean, standard deviation, number, percentage, etc.), analysis of variance (ANOVA), and if necessary, Kruskal–Wallis and Chi-square tests along with charts. Significance level was set at *P* < 0.05.

This study was approved by the Ethics Committee of Ahvaz Jundishapur University of Medical Sciences (Ref. ID.: IR.AJUMS.REC.1399.157) and registered in the Iranian Registry for Clinical Trials (Ref. ID.: IRCT20200513047430N1).

### Results

**Fig. 1:**
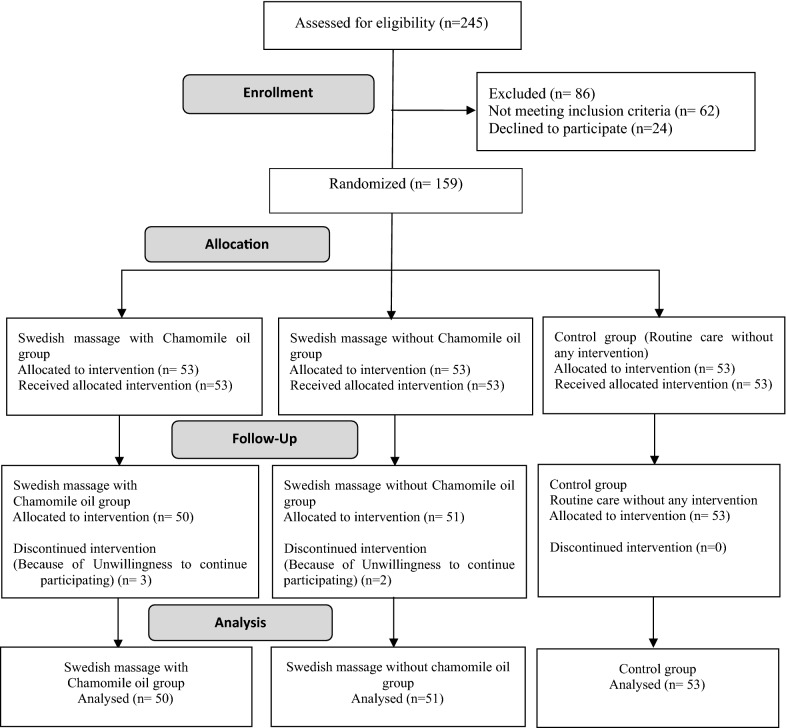
Flowchart of the study

At the end of the study, there were 53 women in the group receiving Swedish massage with chamomile oil group, 51 women in the group receiving Swedish massage without chamomile oil, and 50 women in the control group (Fig. 1).

The results showed that the mean age of participants was 21.98 ± 3.69 in the group receiving Swedish massage with chamomile oil, 22.82 ± 3.48 in the group receiving Swedish massage without chamomile oil, and 23 ± 3.12 in the control group. The mean gestational age in the three groups was 39.15 ± 1.08, 39.29 ± 1.04 and 38.82 ± 0.90 weeks, respectively. The three groups were homogeneous in terms of demographic characteristics, and no statistically significant difference was found between them (*P* > 0.05) (Table 1).

**Table 1:** Comparison of the mean of demographic variables of primiparous women in the three groups studied

	Group			
	Swedish massage with chamomile oil (*n* = 53)	Swedish massage without chamomile oil (*n* = 51)	Control (*n* = 50)	*P*-value
Variable				
Age (years); (Mean ± SD)	21.98 ± 3.69	22.82 ± 3.48	23 ± 3.12	0.277^*^
Gestational age (weeks); (Mean ± SD)	39.15 ± 1.08	39.29 ± 1.04	38.82 ± 0.90	0.057^*^
BMI prior to pregnancy; (Mean ± SD)	21.45 ± 1.64	20.89 ± 1.23	21.27 ± 1.41	0.134^*^
Educational attainment; n (%)				0.057^**^
Primary	19 (35.8)	12 (23.5)	8 (16)	
Junior High school	12 (22.6)	5 (9.8)	6 (12)	
High school diploma	19 (35.8)	28 (54.9)	30 (60)	
University degree	3 (5.7)	6 (11.8)	6 (12)	
Occupation; n (%)				0.660^**^
Employed	7 (13.2)	5 (9.8)	8 (16)	
Housewife	46 (86.8)	46 (90.2)	42 (84)	

* ANOVA ** Chi-square

There was no statistically significant difference between the three groups in terms of pain intensity in 5 cm dilatation (*P*-value = 0.530), but a statistically significant difference was observed between the three groups in terms of pain intensity in the active phase and the second stage of labor (*P*-value < 0.001). The lowest intensity of labor pain was observed in the Swedish massage group with chamomile oil (Table 2).

**Table 2:** Comparison of mean pain intensity based on VAS in primiparous women in the three groups studied

	Group			
Variable	Swedish massage with chamomile oil (*n* = 53)	Swedish massage without chamomile oil (*n* = 51)	Control (*n* = 50)	*P*-value
Before intervention (Mean ± SD)				
Pain intensity at 5 cm dilation	5.28 ± 0.95	5.06 ± 0.97	5.25 ± 1.05	0.530^*^
After intervention (Mean ± SD)				
Pain intensity at 8 cm dilation (active phase)	5.30 ± 0.91	5.67 ± 0.76	6.58 ± 1.02	< 0.001^*^
Pain intensity at 10 cm dilation (active phase)	6.18 ± 0.96	6.61 ± 1.02	7.89 ± 0.71	< 0.001^*^
Pain intensity at second stage of labor	7.12 ± 1.02	7.72 ± 1.07	8.83 ± 0.74	< 0.001^*^

* ANOVA

**Fig. 2:**
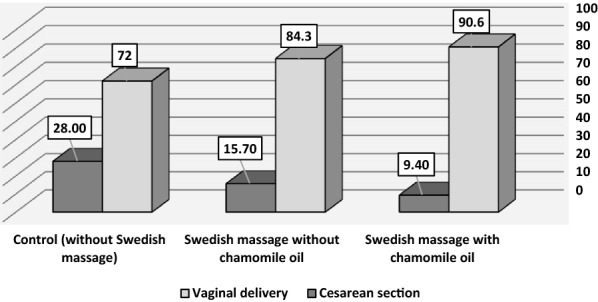
Comparison of frequency (percentage) type of delivery of women in the three groups studied

In this study, a significant difference was observed in terms of the type of delivery between the intervention and control groups (*P* < 0.001) (Table 3; Fig 2 ). Also, there was a statistically significant difference between the three groups in terms of maternal satisfaction with the delivery process (*P* < 0.001). The highest level of satisfaction was observed in the group receiving Swedish massage with chamomile oil.

**Fig. 3:**
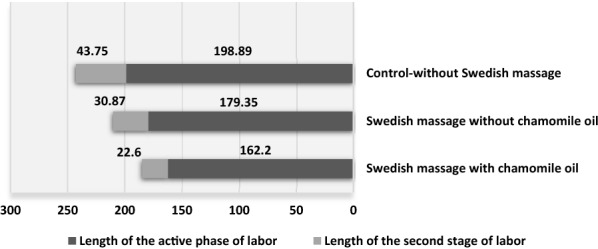
Comparison of mean length of active phase and second stage of labor in the three groups studied

Also, there was a statistically significant difference between the three groups in terms of the length of the active phase and the second stage of labor (*P* < 0.05), (Table 3; Fig. 3), but no statistically significant difference was found between the three groups in terms of first- and fifth-minute Apgar scores (*P* > 0.05) (Table 3).

**Table 3:** Comparison of mean satisfaction score, length of the first and second stage of labor, Apgar score, and type of delivery of primiparous women in the three groups studied

Variable	Group			
	Swedish massage with chamomile oil (*n* = 53)	Swedish massage without chamomile oil (*n* = 51)	Control (*n* = 50)	*P*-value
Satisfaction of childbirth process	68.94 ± 5.24	63.35 ± 5.41	45.08 ± 6.26	< 0.001^*^
Length of the first stage of labor (minutes)	162.20 ± 45.85	179.35 ± 46.24	198.89 ± 63.02	0.006^*^
Length of the second stage of labor (minutes)	22.60 ± 9.91	30.87 ± 12.26	43.75 ± 22.02	< 0.001^*^
First-minute Apgar score	8.96 ± 0.20	8.96 ± 0.21	8.83 ± 0.45	0.091^*^
Fifth-minute Apgar score	10 ± 00	10 ± 00	9.97 ± 0.17	0.265^*^
Type of delivery; %				< 0.043^**^
Normal	48 (90.6)	43 (84.3)	36 (72)	
Cesarean	5 (9.4)	8 (15.7)	14 (28)	

* Kruskal–Wallis ** Chi-square test

### Discussion

The aim of this study was to determine the effect of Swedish massage with (out) chamomile oil on the outcomes of childbirth and the level of satisfaction of primiparous women in Masjed Soleiman, southwest of Iran. The results showed that the mean score of labor pain intensity in the active phase and the second stage of labor was significantly lower in the group receiving chamomile oil massage compared to the one receiving Swedish massage without chamomile oil and the control group.

Massage has been reported to stimulate the nerve fibers, which in turn prevents the transmission of painful stimuli caused by uterine contractions (gate control theory) and thus relieves pain. Massage also increases the threshold of pain in the fetus [7]. In a systematic review study conducted by Ranjbaran et al., massage was recommended to effectively relieve labor pain [28]. Widyawati et al. studied the effect of abdominal and Effleurage massage on labor pain, and showed that Effleurage massage, which is one of the techniques used in Swedish massage, can significantly reduce labor pain in pregnant women and its effectiveness depends on various factors including gestational age, duration of massage, and the amount of pressure applied to the position receiving the massage [29]. Noorollahi et al. compared the effects of massage, hot compress, and routine perineal care on perceived stress and labor pain intensity in primiparous mothers in Iran. Their results showed a greater reduction in pain intensity in the massage therapy group compared to the hot compress group, while the routine perineal care group experienced no significant difference before and after the intervention [30].

In another study conducted by Kaçar et al., massage significantly reduced the pain score (measured by VAS) in the intervention group compared to the control group [31]. Yousefzadeh et al. reported that massage with ginger oil brings about pain relief in the active phase of labor until the end of the active phase [32]. Results of Seri Valan et al. showed that Effleurage and abdominal massage are effective, safe and practical ways to reduce pain in the third trimester of pregnancy and childbirth [33]. Studies also show that oil massage is more effective in controlling pain than massage alone. Yousefzadeh et al., for example, showed that lumbar massage with ginger oil compared to oil-free massage is significantly more effective in reducing childbirth pain in the active phase and leads to an increase in the mothers' satisfaction with the delivery process [32]. In another study by Sritoomma et al., the use of aromatic oils in combination with Swedish massage was shown to increase the effectiveness of these techniques [34]. Alavi Fili et al.'s study also showed that massage with jasmine oil had a greater effect on reducing labor pain in all stages of labor, compared to aromatherapy with jasmine oil and routine care [20]. The results of Hosseini et al.'s study showed that massage therapy with almond and lavender oils significantly reduced the length of labor compared to the control group and that massage therapy could reduce maternal pain and anxiety during labor by reducing the length of the first and second stages of labor [35].

Since no study has yet been conducted on the effect of massage with chamomile oil in reducing labor pain, here we cite studies that have dealt with the analgesic properties of this plant. The results of Ameri et al., for instance, showed that massage with chamomile and lavender oils reduces the severity of pain in patients since the aromatic and volatile oils of these plants are gradually absorbed through the skin (between 10 and 30 min), applying their herbal therapeutic effects such as sedative, analgesic, anti-contraction, and anti-cramping [23].

One of the problems that mothers have to deal with during childbirth is prolonged childbirth. It is associated with adverse maternal and fetal complications and is a period replete with worry and anxiety. This condition is also accompanied by stimulation of the secretion of stress hormones, especially cortisol, reduced energy, and increased maternal fatigue, which increases the duration of labor and makes the mother less cooperative during labor [35]. In their study on the effect of massage on pain and duration of labor, Field et al. found that massage reduced pain and duration of labor [36]. Abbasi et al. also found that the mean length of delivery in the first and second stages in the group receiving massage was significantly lower than that in the control group [37]. In the study of Chuntharapat et al., the duration of the first and second stages of labor in the intervention group was significantly reduced [38]. Khodakarami et al. reported that the duration of the first stage of labor was significantly shorter in the group receiving Swedish massage compared to the control group [39]. Another study by Gönenç et al. revealed that the duration of the first stage of labor was shortest (245 min) in the massage therapy group alone and longest (350 min) in the control group [40].

Haghighi et al. found that massage therapy during childbirth shortens the length of the first and second stages of labor and improves first- and fifth-minute Apgar scores. As a result, by shortening the duration of labor, pregnant women will be more willing to give birth naturally [16].

Our results also showed that in the two groups undergoing intervention with Swedish massage, the rate of normal delivery was higher than that of cesarean delivery. Gallo et al. investigated the effect of massage on reducing the severity of labor pain in 46 mothers in two groups of massage therapy (*n* = 23) and control (*n* = 23). The subjects in the intervention group received back massage at points T10 to S4 for 30 min in the active phase of labor (dilation of 4–5 cm) and the pain and outcomes of labor were evaluated. The results showed that in the intervention group, 74% had normal delivery and 26% had cesarean section. In the control group, on the other hand, 83% of the mothers had a normal delivery as opposed to 17% who went through a cesarean section [25]. Since in the present study the rate of natural childbirth in the massage group was higher than that of cesarean section, Gallo et al.'s results are in line with ours.

In the present study, no statistically significant was found between first- and fifth-minute Apgar scores in the three groups. This is consistent with the results of Neetu et al. who found no statistically significant difference between first- and fifth-minute Apgar scores in the two groups studied with the aim of investigating the effect of effleurage abdominal massage on the severity of labor pain and delivery outcomes in primiparous women [41].

In contrast, Chang et al. who studied the effect of massage on labor pain and anxiety during childbirth found no significant difference between the massage therapy and control groups, and only a slight increase in the first stage of labor was reported in the massage therapy group [42]. The reason for this discrepancy in results could be attributed to the fact that in the study of Chang et al., massage techniques were performed by husbands while in our study, the Swedish massage techniques were performed by a trained midwife. Field et al. reported that the massage techniques carried out by the doula were more effective than those performed by the husband [36]. Janssen et al.'s results confirmed the effectiveness of the Swedish massage techniques in reducing labor pain, yet no statistically significant difference was found in reducing the duration of the first stage of labor [7]. This difference in results can be explained by the fact that they used no massage oil in their study and that we used several Swedish massage techniques in our study.

One of the limitations of this study which was beyond the researchers' control was the increased fear and anxiety of pregnant mothers participating in the study due to the Covid-19 pandemic, which might have affected the results. Future studies are recommended to be performed with a larger sample size and to examine the effect of Swedish massage techniques on other delivery outcomes.

### Conclusion

The results of the study showed that using Swedish massage techniques alone or in combination with chamomile oil can improve the outcomes of labor such as reduced pain, shorter duration of labor, increased labor satisfaction, and a higher rate of normal delivery as opposed to cesarean section among primiparous women. Therefore, given its lower side effects and cost compared to therapeutic analgesic methods, massage with chamomile oil can be considered as one of the complementary medicine methods to reduce pain in women during different stages of labor.

## Data Availability

The datasets generated and/or analyzed during the current research are not publicly available as individual privacy could be compromised but are available from the corresponding author on reasonable request.
